# Limitations of GPT‐4 in analyzing real‐life medical notes related to cognitive impairment

**DOI:** 10.1111/psyg.13002

**Published:** 2023-06-27

**Authors:** Yat‐fung Shea, Nok‐Yee Charlotte Ma

**Affiliations:** ^1^ Department of Medicine, Queen Mary Hospital University of Hong Kong Pokfulam Hong Kong; ^2^ Faculty of Medicine University of Hong Kong Pokfulam Hong Kong

Dear Editor,

There has been a surge of interest in using artificial intelligence (AI) in medical consultation, for example, drug interactions or medical images analyses.^1^ One type of AI is the AI chatbot. GPT‐4 (Generative Pre‐trained Transformer 4) is an AI system with chat interface.^1^ A ‘session’ is started by entering a query and then ‘response’ in natural‐language will be given within a second. The system is able to keep track of the context of the ongoing conversation so that the conversation will be more useful and natural. Patients with cognitive impairment often have long medical histories about their presentation, and also clinicians will need to differentiate between different types of dementia, for example, Alzheimer's disease (AD) and frontotemporal dementia (FTD), with overlapping features. Subtle details may be missed by clinicians and AI may be able to pick up these subtle features to alert clinicians on the most likely diagnoses. We have explored on whether GPT‐4 can help clinicians in making the most probable diagnoses of cognitive impairment.

The medical histories were retrieved from the electronic medical record system of patients managed in the Memory Clinic, Queen Mary Hospital in Hong Kong between 2008 and 2023 (except one patient managed as an in‐patient, patient 6) (Table 1 and Supplementary file 1 in Data S1). We have chosen 10 representative medical histories that are commonly encountered in the Memory Clinic or are usually referred for further advice. The medical histories were extracted from medical notes just before further investigations were ordered (Supplementary file 1 in Data S1). All the patients had been managed by a clinician with over 15 years of clinical experience in the Memory Clinic. All the patients had their underlying causes of cognitive impairment diagnosed using genetic testing, neuroimaging or sleep study according to latest clinical diagnostic guidelines.^2^, ^3^, ^4^, ^5^ Two cases were previously reported.^6^, ^7^ The medical histories were entered into GPT‐4 on the same day with exact script copied out and further analyzed (Supplementary file 1 in Data S1). We extracted the primary diagnosis, differential diagnoses and work‐up as suggested by GPT‐4 and compared these with those documented by the clinician.

**Table 1:** Summary of responses of GPT‐4 and clinicians in 10 real‐life scenarios of patients with cognitive impairment

Scenarios	Final diagnosis (supportive investigation findings, details refer to Supplementary file 2 in Data S2)	GPT‐4 (1st dx correctness = 3/10; if include ddx & work‐up that may lead to correct dx = 7/10)	Clinician's documentation (1st dx correctness = 9/10)
Patient 1	CADASIL causing vascular dementia and parkinsonism (genetic testing showed heterozygous mutation with a pathogenic variant c.1630C > T, p.Arg544Cys of NOTCH3 gene)	1st dx: vascular dementia Ddx: AD, LBD, NPH, FTD, PDD Work‐up suggested: MRI brain, neuropsychological testing, genetic test (not specified), referral to neurologist or geriatrician	1st dx: CADASIL causing vascular cognitive impairment and parkinsonism Ddx: None Work‐up suggested: NOTCH3 gene mutation testing after genetic counselling
Patient 2	CBS (MRI brain showed left perirolandic atrophy)	1st dx: PSP Ddx: PDD, CBS, vascular parkinsonism Work‐up suggested: MRI brain, neuropsychological testing, autonomic function test, neurologist referral	1st dx: CBS Ddx: PSP Work‐up suggested: MRI brain
Patient 3	Semantic dementia (^18^FDG‐PET showed bilateral anterior temporal lobes hypometabolism and amyloid PET scan using ^11^C‐PiB negative)	1st dx: AD Ddx: VAD, FTD, LBD, NPH, vitamin B12 or folate deficiency Work‐up suggested: CSF AD biomarkers, MRI brain, neuropsychological testing, referral to neurologist or geriatric psychiatrist	1st dx: AD Ddx: PPA‐FTD Work‐up suggested: ^18^FDG‐PET and amyloid PET, CSF AD biomarkers
Patient 4	CBS (MRI brain showed right perirolandic atrophy)	1st dx: CBS Ddx: PDD, PSP, MSA, vascular dementia Work‐up suggested: MRI brain, DaTscan, referral to neurologist	1st dx: CBS Ddx: PSP Work‐up suggested: MRI brain
Patient 5	PCA (amyloid scan positive using ^11^C‐PiB and MRI brain showed bilateral occipital lobes atrophy)	1st dx: PPA Ddx: AD, VAD, bvFTD, PDD Work‐up suggested: MRI brain, neuropsychological testing, CSF AD biomarkers, refer to neurologist, genetic test if strong family history	1st dx: PCA Ddx: semantic dementia Work‐up suggested: ^18^FDG‐PET, amyloid PET, CSF AD biomarkers
Patient 6	CJD (MRI showed multiple areas of gyral restricted diffusion; EEG showed generalized periodic sharp waves with occasional triphasic morphology)	1st dx: dementia (not further specified) Ddx: depression, delirium, nutritional deficiencies, NPH Work‐up suggested: neuropsychological testing, EEG, MRI brain, medication review, psychiatrist consultation for e.g. depression	1st dx: CJD Ddx: non‐convulsive status epilepticus Work‐up suggested: MRI brain, EEG
Patient 7	OSA (respiratory disturbance index 43.4/h during polysomnography)	1st dx: amnesic MCI Ddx: AD, VAD, sleep apnoea, depression Work‐up suggested: neuropsychological testing, MRI brain/PET, sleep study	1st dx: OSA Ddx: depression Work‐up suggested: polysomnography
Patient 8	AD (reduced Aβ‐42, elevated total tau and p‐tau)	1st dx: AD Ddx: VAD, LBD, bvFTD, NPH, depression Work‐up suggested: MRI brain; ^18^FDG‐PET, CSF AD biomarkers, genetic testing if strong family history, referral to neurologist/geriatrician	1st dx: AD Ddx: semantic dementia Work‐up suggested: ^18^FDG‐PET +/− amyloid PET, CSF AD biomarkers
Patient 9	DLB (^18^FDG‐PET showing bilateral parieto‐occipital hypometabolism and negative amyloid PET using ^11^C‐ PiB)	1st dx: LBD Ddx: AD, PDD, delirium, VAD, NPH Work‐up suggested: neuropsychological testing, MRI brain; EEG, polysomnography, dopamine transporter, lumbar puncture, referral to neurologist/geriatrician	1st dx: DLB Ddx: AD Work‐up suggested: ^18^FDG‐PET/SPECT brain
Patient 10	bvFTD (SPECT brain showed bilateral frontal lobes and temporal lobes hypoperfusion)	1st dx: AD Ddx: VAD, bvFTD, NPH, MCI Work‐up suggested: neuropsychological testing, MRI brain, ^18^FDG‐PET, CSF AD biomarkers, referral to neurologist/geriatrician/psychiatrist	1st dx: bvFTD Ddx: AD Work‐up suggested: ^18^FDG‐PET/SPECT brain

Abbreviations: AD, Alzheimer's disease; bvFTD, behavioural variant frontotemporal dementia; CADASIL, cerebral arteriopathy with subcortical infarcts and leukoencephalopathy; CBS, corticobasal syndrome; CJD, Creutzfeldt‐Jakob disease; CSF biomarkers, cerebrospinal fluid biomarkers (including Aβ‐42, total tau and p‐tau); DLB, dementia with Lewy bodies; dx, diagnosis; Ddx, differential diagnoses; EEG, electroencephalograph; ^18^FDG, fluorodeoxyglucose; FTD, frontotemporal dementia; FAD, familial Alzheimer's disease; LBD, Lewy body dementia; MCI, mild cognitive impairment; MRI, magnetic resonance imaging; NOTCH3, neurogenic locus notch homologue protein 3; NPH, normal pressure hydrocephalus; MSA, multiple system atrophy; OSA, obstructive sleep apnoea; PCA, posterior cortical atrophy; PD, Parkinson's disease; PDD, Parkinson's disease dementia; PiB, Pittsburgh Compound B; PPA, primary progressive aphasia; PSP, progressive supranuclear palsy; SPECT, single photon emission computed tomography; VAD, vascular dementia.

The results are shown in Table 1. The overall accuracy in making the primary diagnosis was 30% for GPT‐4 and 90% for our clinician. If one followed the work‐up or considered the differential diagnoses as suggested by GPT‐4, four more correct diagnoses should be able to be made (i.e., 70%). There were several limitations immediately evident from the results. First, GPT‐4 had not suggested the usage of amyloid positron emission tomography. Second, GPT‐4 had problems in correlating the medical histories with the provided neuroimaging findings (patient 2, absence of midbrain atrophy) and would suggest the same neuroimaging method (patient 1) or blood tests (patient 10). Third, GPT‐4 was unable to pick up certain subtle clinical features, for example, strong family history of young onset stroke (patient 1), homonymous hemianopia (patient 5), obesity (patient 7) and stereotyped behaviour (patient 10). However, GPT‐4 was able to diagnose patients with typical presentations of AD (patient 8) and dementia with Lewy bodies (patient 9). GPT‐4 could give a general comprehensive follow‐up plan, for example, referral to multiple disciplinary teams but not a more focused suggestion resulting in redundant work‐up.

The advantage of our study was that we had tried to include a range of presentations of cognitive impairment, from typical to atypical, from rapidly progressive dementia to slowly progressive dementia and including rare clinical scenarios. The limitation of our study was that we did not have post‐mortem neuropathological diagnoses and the number of medical histories analyzed was small. We do not know whether recording the exact wordings of clinical consultation between the doctors and the patients or feeding in clinical information step by step may generate a different outcome.

Overall, we would not suggest clinicians rely on GPT‐4 in managing patients with cognitive impairment. Entering the wrong clinical or radiological information into GPT‐4 will result in wrong interpretation and formulation of incorrect plans. The wrong primary diagnosis may mislead the clinical management and may result in medical litigation. GPT‐4, at the moment, is not able to help clinicians to pick up some subtle clinical details. We recommend a re‐testing of GPT‐4 when a newer version is available. It is important for the medical community to monitor the clinical accuracy of any AI system so as to avoid misuse.

## Disclosure

The authors declare they have no conflicts of interest in the research.

## Supporting information


**Data S1.** Supporting information.

**Data S2.** The neuroimaging findings supporting the diagnoses of our cases series.
