# Supplementary material for: Limitations of GPT‐4 in analyzing real‐life medical notes related to cognitive impairment
Source: Psychogeriatrics. 2023 Jun 27;23(5):885–7. doi: 10.1111/psyg.13002 (PMC11577998; doi:10.1111/psyg.13002)
Supplement: Supplementary file 1 — Data S1. Supporting information. [file PSYG-23-885-s002.docx]

**Supplementary file 1**

**Patient 1**

For the following medical history, we would like to ask you some questions at the end.

A 62 year-old man was referred for stepwise deterioration in memory. The patient was noted to have short term memory impairment with stepwise deterioration since six months ago. He repeatedly retrieved money from ATM machine. He forgot to switch off light. He forgot to switch off the water-tap. He forgot that he has cooked food. His remote memory was relatively preserved. There was no prosopagnosia or anomia. There was no rapid eye movement sleep behavioral disorder, vivid visual hallucination, stereotyped behavior, hoarding behavior, or disinhibition.

The patient had judgement problem. He had walking instability and weakness suddenly. There was temporal and spatial disorientation. The hygiene was very poor at home with rotten food and also with insects at home. He needed close supervision for medications. Meal was prepared by maid or close relatives. He can walk two block length. He cannot use the correct utensil. But he can use his phone, and What's app. He had urinary incontinence.

He had history of hypertension, impaired fasting glucose, presbycusis and atypical chest pain with normal exercise tolerance. He had three episodes of stroke since the age of 50 years old. These episodes presented as left lower limb monoplegia, left sided hemiplegia and slurring of speech 12 years, 8 years and 1 year ago respectively.  Extensive work-up including 24-hour Holter monitoring and transthoracic echocardiogram were unremarkable. He has multiple first degree relatives, including his brother, suffering from stroke in their fifties.  Twelve years ago, magnetic resonance imaging of the brain reportedly showed bilateral carotid plaques, multiple cerebral infarctions, acute cerebral infarction noted in the posterior limb of the right internal capsule; multiple chronic lacunar infarct noted in the deep subcortical white matter and the right basal ganglion. He has no known drug allergy. He is an ex-smoker and non-drinker. He received up to university level of education. He previously worked as a social worker. One year ago, magnetic resonance imaging showed evidence of hemorrhagic infarction over the bilateral external capsule; established infarctions at bilateral basal ganglia, left thalamus and pons.

Physical examination reviewed: blood pressure was 146/77 mmHg and pulse was 95 bpm. Upon inspection of his gait, he showed loss of arm swing, small steps with stooped posture. There was no carotid bruits, no eye blinking and with positive glabellar tap. Bilateral extremities showed mild increase in rigidity but there was no resting tremor. Limbs power were normal. Cardiovascular and respiratory examination were unremarkable.  His Lawton activities of daily living score was 3 out of 5; simplified Barthel index was 15 out of 20; Neuropsychiatric Inventory score was 6 out of 120 (scoring 1 for dysphoria, 1 for apathy; 4 for irritability/ lability; Hong Kong Version of Montreal Cognitive Score was 10 out of 30; geriatric depression score was 2 out of 15. Vitamin B12, folate, thyroid function test and Venereal Disease Research Laboratory test were unremarkable. The latest computed tomography of the brain showed also extensive periventricular hypodensities.

Based on the above,

1. What is your primary diagnosis?
2. What are your differential diagnoses?
3. How would you work up for this patient?

**GPT-4 reply:**

1. Primary Diagnosis:

Based on the provided history, the primary diagnosis for this patient is likely Vascular Dementia. This is supported by the history of multiple strokes, cognitive decline with stepwise deterioration, and imaging findings of multiple cerebral infarctions and lacunar infarcts.

2. Differential Diagnoses:

- Alzheimer's disease: Although less likely due to the stepwise deterioration and history of multiple strokes, Alzheimer's disease could still be considered in the differential diagnosis.

- Lewy Body Dementia: The patient does not present with rapid eye movement sleep behavioral disorder or vivid visual hallucinations, which are characteristic features of Lewy Body Dementia, but it could still be considered as a differential diagnosis.

- Normal pressure hydrocephalus: This condition could also present with gait disturbances, cognitive decline, and urinary incontinence; however, the patient's history of multiple strokes makes vascular dementia a more likely diagnosis.

- Frontotemporal Dementia: The patient does not present with stereotyped behavior, hoarding behavior, or disinhibition, which are characteristic features of Frontotemporal Dementia, but it could still be considered as a differential diagnosis.

- Parkinson's disease dementia: The patient does have some parkinsonian features, such as loss of arm swing and rigidity, but there is no resting tremor.

3. Workup for this patient:

- Detailed neurological examination: To evaluate the patient's cognitive and motor functions more thoroughly.

- Neuroimaging: A repeat MRI of the brain could provide more information about the extent of the patient's cerebrovascular disease and help rule out other possible diagnoses.

- Neuropsychological testing: Comprehensive neuropsychological testing can help characterize the patient's cognitive deficits and assist in diagnosis.

- Blood tests: Complete blood count, metabolic panel, and inflammatory markers can help identify possible contributing factors or rule out other underlying conditions.

- Genetic testing: If a strong family history of early-onset dementia is suspected, genetic testing might help identify a hereditary component.

- Referral to a specialist: A neurologist or geriatrician with expertise in dementia should evaluate the patient for a more accurate diagnosis and management plan.

Follow up question to GPT-4: “Are you aware of any genetic disease that we may need to investigate?”

While vascular dementia is not typically considered a genetic disease, some genetic factors can increase the risk of developing vascular problems that may contribute to dementia. In this case, given the patient's multiple first-degree relatives suffering from strokes in their fifties, it is worth considering the possibility of a genetic predisposition to cerebrovascular disease.

Some genetic conditions to consider include:

1. Cerebral Autosomal Dominant Arteriopathy with Subcortical Infarcts and Leukoencephalopathy (CADASIL): This is a rare genetic disorder caused by mutations in the NOTCH3 gene, leading to recurrent strokes and cognitive decline.

2. Hereditary cerebral amyloid angiopathy (HCAA): This condition is associated with mutations in the APP, CST3, or ITM2B genes and results in the deposition of amyloid proteins in cerebral blood vessels, increasing the risk of hemorrhagic strokes and cognitive decline.

3. Familial hypercholesterolemia: This genetic condition is caused by mutations in the LDLR, APOB, or PCSK9 genes and results in high cholesterol levels, which can increase the risk of atherosclerosis and strokes.

To investigate these genetic diseases, a detailed family history should be obtained, focusing on the presence of cerebrovascular disease, cognitive decline, and the age of onset for these conditions. Based on the family history and the patient's clinical presentation, genetic testing for specific gene mutations may be considered in consultation with a genetic counselor or specialist. Keep in mind that genetic testing may have ethical and emotional implications for the patient and their family members, so it's essential to discuss these factors before proceeding with any genetic testing.

**Patient 2**

For the following medical history, we would like to ask you some questions at the end.

A 77 year-old man was referred for atypical parkinsonism.

Family noticed the patient to have evidence of short term memory decline and poor recall of recent events seven years ago. He also had repetitive conversations, forgot recent instructions, and kept topping up the value within octopus travel card. The memory progressively declined and he started to forget to turn off the water tap, forgot to bring the front door key when went out, and overcooked food. He started to get lost in familiar place four years ago. He would urinate in the garden, or urinate in a plastic bag four years ago. He went downstairs with his shoes, threw the shoes away and walked back with bare food. He was noted to have decline in activities of daily living, bleeding while shaving, and with deteriorated personal hygiene. However, he still remembered grandchildren's names. He threw rubbish via the window in secret when others were not looking or asleep. Over the last few months, he felt weak and unable to shave himself.

He presented with three years history of walking with small steps and suffered from an episode of fall two years ago and ended up with left neck of femur fracture requiring operation. He has been observed to have more significant left upper limb rigidity. Daughter observed delayed in response to pain after scalded injury. The patient has dysphasia with non-fluent speech for around 1 year. The patient had anomia. There was no REM sleep behavioral disorder or vivid visual hallucination. There was a single episode of syncope. There was no fluctuation of cognitive function. There was no stereotyped behavior. Madopar has been started before with poor response in mobility. Basic activities of daily living are dependent. He has been observed before to have throwing limbs and then removing the things on the table involuntarily. He was an old age home resident and walked with a frame.

Past medical history includes osteoarthritis of knee joints with bilateral total knee replacement, history of scalded injury to the right medial thigh with persistent pain, iatrogenic Cushing’s syndrome, hypertension, benign prostatic hyperplasia and paroxysmal atrial fibrillation on apixaban.

Physical examination noted blood pressure of 97/61 mmHg and pulse rate of 73 bpm. Upper limb examination showed left sided rigidity more than right side. The glabellar tap was positive. There was evidence of vertical gaze palsy. Cardiovascular and respiratory examination was unremarkable. Palmomental reflexes were positive. Hong Kong version of the Montreal Cognitive Assessment was 6 out of 30. Vitamin B12, folate, thyroid function test and Venereal Disease Research Laboratory were negative. Computed tomography of the brain was described as generalized cerebral atrophy. Previous magnetic resonance imaging showed no evidence of midbrain atrophy.

Based on the above,

1. What is your primary diagnosis?
2. What are your differential diagnoses?
3. How would you work up for this patient?

GPT-4 reply:

1. Primary Diagnosis:

Based on the provided history and examination findings, the primary diagnosis for this patient is likely Progressive Supranuclear Palsy (PSP). This is supported by the patient's age, the presence of vertical gaze palsy, atypical parkinsonism with poor response to levodopa, and cognitive decline.

2. Differential Diagnoses:

- Parkinson's disease dementia: Although the patient had poor response to levodopa, Parkinson's disease dementia could still be considered due to the presence of parkinsonian features and cognitive decline.

- Corticobasal degeneration: This condition could present with asymmetric rigidity, cognitive decline, and apraxia, which might be consistent with the patient's presentation.

- Multiple system atrophy: This condition can present with parkinsonism, autonomic dysfunction, and cerebellar signs. The patient's history does not mention significant autonomic symptoms, but it could still be considered as a differential diagnosis.

- Vascular parkinsonism: Given the patient's age and history of vascular risk factors, vascular parkinsonism could be considered as an alternative diagnosis.

3. Workup for this patient:

- Detailed neurological examination: To evaluate the patient's motor, cognitive, and autonomic functions more thoroughly.

- Neuroimaging: A repeat MRI of the brain, including specialized sequences to evaluate the midbrain and other structures, could provide more information about the patient's condition and help confirm the diagnosis or identify alternative diagnoses.

- Neuropsychological testing: Comprehensive neuropsychological testing can help characterize the patient's cognitive deficits and assist in diagnosis.

- Autonomic function tests: If there is a suspicion of multiple system atrophy or other conditions with autonomic dysfunction, these tests can help evaluate the patient's autonomic function.

- Referral to a specialist: A neurologist with expertise in movement disorders should evaluate the patient for a more accurate diagnosis and management plan.

**Patient 3**

For the following medical history, we would like to ask you some questions at the end.

A 67 year-old man was referred to our memory clinic with progressive memory impairment and language impairment.

The patient was noted to have progressive impairment in short term memory for four years including inability to recall what food he has taken for breakfast and he was unable to recall the mode of transport he used to come to the clinic. The long term memory was preserved. He also had difficulty in naming objects over the past four years and he was also unable to write. He has impairment in calculation.  He has difficulty in recognizing less often seen relatives. He had temporal disorientation. However, he was still able to recognize the places near to his home. He was unable to manage his own finances but he could manage to go to the toilet and take a bath alone. There was no evidence of hallucination and delusion.

He has no known drug allergy. He is an ex-drinker. He lived with his daughter and wife. He received education up to primary level six. He previously ran a shipping company and has retired. He was able to walk with a stick. He had a history of alcohol related bilateral avascular necrosis of the hip with bilateral total joint replacement performed for both hip joints.

Physical examination reviewed inability to write (dyslexia), inability to name less often seen objects e.g. stethoscope, walking aid. He was able to name the watch and pen. He showed full power on neurological examination without parkinsonism. He was normal in the cardiovascular and respiratory examination. His mini-mental state examination score was 12 out of 30. Geriatric depression scale was 8 out of 30. Neuropsychiatric inventory scale was 19 out of 120. Vitamin B12, folate, Venereal Disease Research Laboratory were normal. Computed tomography of the brain showed generalized cerebral atrophy.

Based on the above,

1. What is your primary diagnosis?
2. What are your differential diagnoses?
3. How would you work up for this patient?

GPT-4 reply:

1. Primary Diagnosis:

Based on the patient's history and examination findings, the primary diagnosis is likely Alzheimer's disease. Alzheimer's is the most common cause of dementia in older adults, and the patient's progressive memory impairment, language difficulties, and difficulty in naming objects are consistent with this diagnosis. The generalized cerebral atrophy seen on the computed tomography (CT) scan of the brain also supports this diagnosis.

2. Differential Diagnoses:

Some differential diagnoses to consider include:

- Vascular dementia: This form of dementia is caused by reduced blood flow to the brain. The patient's history of alcohol-related bilateral avascular necrosis of the hip may suggest a vascular component, but the absence of focal neurological deficits makes this less likely.

- Frontotemporal dementia: This type of dementia is characterized by changes in personality and behavior, as well as language difficulties. However, the patient's age and the nature of his cognitive deficits make Alzheimer's disease more likely.

- Lewy body dementia: This form of dementia is associated with parkinsonism and visual hallucinations. The patient does not exhibit parkinsonism, and there is no evidence of hallucinations or delusions.

- Normal pressure hydrocephalus: This condition can cause cognitive impairment, gait disturbance, and urinary incontinence. However, the patient's CT scan does not show ventricular enlargement, making this diagnosis less likely.

- Vitamin B12 or folate deficiency: These deficiencies can cause cognitive impairment, but the patient's blood tests were normal.

3. Workup for this Patient:

A thorough workup for this patient should include:

- A detailed history and physical examination, focusing on cognitive function, behavioral changes, and neurological symptoms.

- A review of medications and possible drug interactions.

- Laboratory tests to rule out reversible causes of cognitive impairment, including complete blood count, electrolytes, liver function tests, thyroid function tests, and a metabolic panel.

- Cerebrospinal fluid (CSF) analysis to look for biomarkers associated with Alzheimer's disease, such as amyloid-beta and tau protein levels.

- Magnetic resonance imaging (MRI) of the brain to better visualize brain structures and rule out other potential causes of dementia, such as tumors or strokes.

- A neuropsychological assessment to further evaluate the patient's cognitive deficits and determine the extent of impairment.

- Referral to a neurologist or geriatric psychiatrist for further evaluation and management, as appropriate.

**Patient 4**

For the following medical history, we would like to ask you some questions at the end.

A 77 years old woman was referred because of suspected language impairment and suspected Parkinson’s disease dementia.

She was noted to have recurrent fall three years ago with unsteady gait, lower limb weakness and tremor. One episode of fall was suspected to be caused by syncope but with unremarkable work-up on echocardiogram and 24-hour holter examination. Magnetic resonance imaging of the brain showed no evidence of stroke. However, magnetic resonance imaging of the cervical spine showed posterocentral disc protrusion is seen at C2/3 level causing focal impingement on the cervical cord and myelomalacic change is seen from C1-T1  levels. Operation involving excision of intervertebral disc, anterior spinal fusion and excision of bone for graft. Unfortunately the mobility of our patient progressively deteriorated despite offering a course of rehabilitation in geriatric day hospital. She walked with rollator with assistance.

There was no evidence of memory impairment. The patient has preserved spatial and temporal orientation. There was no evidence of rapid eye movement behavior disorder, no vivid visual hallucination, and no evidence of fluctuation of cognitive function or consciousness. The patient was only observed to have some articulation problem. She complained of gradual inability to use her right upper limb with difficulty in dressing. There was also no stereotyped behavior, disinhibition, hypersexuality or low mood. She had history of hypercholesterolemia, essential tremor, osteoporosis with right sided intertrochanteric fracture with operation performed, and right sided thyroid nodule of 1.4 x 1.1 cm in size. She lived with daughter and maid. She needs to walk with one assistant and required assistance in basic activities of daily living.

Physical examination reviewed blood pressure of 150/48 mmHg and pulse of 62 bpm. She was sitting comfortably on wheelchair. She was noted to have marked rigidity and spasticity of right upper limb and dysarthria. Hong Kong Version of Montreal cognitive assessment was 27 out of 30. Vitamin B12, folate, thyroid function test, Venereal Disease Research Laboratory were normal. The mobility of the patient did not improve with madopar 62.5 mg tds po.

Based on the above,

1. What is your primary diagnosis?
2. What are your differential diagnoses?
3. How would you work up for this patient?

GPT-4 reponse:

1. Primary Diagnosis:

Based on the provided medical history, my primary diagnosis for this 77-year-old woman is **corticobasal degeneration (CBD)**. The patient's symptoms include language impairment, unsteady gait, lower limb weakness, tremor, and gradual inability to use her right upper limb. Additionally, she has marked rigidity and spasticity in her right upper limb and dysarthria. These symptoms are consistent with CBD, a progressive neurodegenerative disorder that affects both motor and cognitive functions.

2. Differential Diagnoses:

- Parkinson's disease dementia: The patient presents with suspected Parkinson's disease dementia, but there is no evidence of rapid eye movement behavior disorder, vivid visual hallucinations, or fluctuations in cognitive function or consciousness. Furthermore, the patient's mobility did not improve with madopar, which is commonly used to treat Parkinson's disease symptoms.

- Progressive supranuclear palsy (PSP): PSP is another neurodegenerative disorder that can present with similar symptoms to CBD, such as gait instability, falls, and rigidity. However, the patient's clinical presentation does not include typical features of PSP, such as vertical gaze palsy or specific cognitive deficits.

- Multiple system atrophy (MSA): MSA is a neurodegenerative disorder characterized by autonomic dysfunction, parkinsonism, and cerebellar ataxia. Although the patient has some symptoms consistent with MSA, such as falls and unsteady gait, the lack of autonomic dysfunction and ataxia makes this diagnosis less likely.

- Vascular dementia or multi-infarct dementia: This could be considered given the patient's age and history of hypercholesterolemia. However, the brain MRI showed no evidence of stroke, making this diagnosis less likely.

3. Workup for this patient:

To further investigate the primary diagnosis and rule out differential diagnoses, I would recommend the following tests and consultations:

- A more detailed neuropsychological assessment to better characterize the patient's cognitive and language impairments.

- Neuroimaging studies, including MRI with dedicated sequences to evaluate for signs of CBD, PSP, or other neurodegenerative disorders.

- A DaTscan if clinically indicated, to assess dopaminergic function in the brain and help differentiate between Parkinson's disease and other parkinsonian syndromes.

- A consultation with a movement disorder specialist or neurologist for a more comprehensive evaluation and to discuss potential treatment options for the patient's symptoms.

- Occupational and physical therapy assessments to evaluate the patient's functional abilities and recommend appropriate interventions to improve her quality of life.

**Patient 5**

For the following medical history, we would like to ask you some questions at the end.

A 61 year old man was referred to the memory clinic because of progressive cognitive impairment.
He initially presented with difficulty in writing chinese, including his name for three years. He ignored this as he could still read and write English well. Over the next three years, his wife noticed the patient to have slow speech production and difficulties in reading newspaper and e-mails. Memory, judgment, and executive function started to impair in the last 2 years. He showed risky financial management. He showed repeated questioning and misplaced important items. He missed appointments and had one episode of losing his way. The long term memory history was relatively preserved. There were mild impairment in judgment and time orientation. There was no confabulation, hallucination, delusion or other psychotic features.There was no major change of personality or hypersexuality. But he tended to be more aggressive to his wife, verbally & physically, when confronted with his wife. Socially, he kept playing board games weekly but he noticed his skills had deteriorated. He lived with wife. He is a non-smoker and social drinker. He received up to university level of education. He is a retired accountant. He enjoyed good past health. His mother was diagnosed to have Parkinson’s disease in her 80s.

Neurological examination was unremarkable except examination of visual field noted right homonymous hemianopia. The patient showed no problem in verbal comprehension, repetition, or fluency. He had difficulty in naming details of objects. He was unable to write Chinese. He showed dyslexia and dysgraphia. His Mini-Mental State Examination score was 25 out of 30. Hong Kong version of Montreal Cognitive Assessment was 24 out of 30.  Vitamin B12, folate, thyroid function test and Venereal Disease Research Laboratory was unremarkable. Computed tomography of the brain did not note any evidence of infarct.

Based on the above,

1. What is your primary diagnosis?
2. What are your differential diagnoses?
3. How would you work up for this patient?

GPT-4 response:

1. Primary Diagnosis:

Based on the provided history and examination findings, the most likely primary diagnosis for this patient is a form of neurodegenerative dementia, specifically, Primary Progressive Aphasia (PPA). The patient's progressive difficulty in writing Chinese, slow speech production, difficulties in reading, and cognitive decline over time are characteristic features of PPA. Additionally, the patient's relatively preserved long-term memory and verbal comprehension, repetition, and fluency are consistent with this diagnosis.

2. Differential Diagnoses:

- Alzheimer's disease: Although PPA is a variant of Alzheimer's disease, the more classic presentation of Alzheimer's typically involves memory loss as the initial and most prominent symptom. However, Alzheimer's cannot be ruled out completely, as some patients may present with atypical symptoms.

- Vascular dementia: The presence of right homonymous hemianopia suggests the possibility of an underlying cerebrovascular event. However, the CT scan did not reveal any evidence of infarct.

- Frontotemporal dementia: Some symptoms, such as impaired judgment and changes in social behavior, overlap with those of frontotemporal dementia. However, the language and speech difficulties described in this case are more suggestive of PPA.

- Parkinson's disease dementia: Given the family history of Parkinson's disease, it is worth considering. However, the patient's symptoms are more consistent with PPA, and there is no mention of motor symptoms typical of Parkinson's disease.

3. Workup for this patient:

Further evaluation and workup for this patient may include:

- Magnetic resonance imaging (MRI) of the brain: To better evaluate for any structural abnormalities, atrophy, or vascular changes that may not have been apparent on the CT scan.

- Neuropsychological assessment: To obtain a more detailed evaluation of the patient's cognitive abilities, language function, and other cognitive domains.

- Cerebrospinal fluid (CSF) analysis: To examine biomarkers for Alzheimer's disease, such as amyloid-beta and tau proteins, which may help to confirm or rule out Alzheimer's disease as a cause of the patient's symptoms.

- Genetic testing: In some cases, testing for mutations associated with familial forms of dementia may be appropriate.

- Consultation with a neurologist or specialist in memory disorders: To further evaluate the patient's symptoms and guide the diagnostic process.

Throughout the workup, it is essential to monitor the patient's symptoms and involve their family in the process to help provide support and gather additional information about the patient's history and functioning.

Follow-up question: “Are you aware any form of atypical Alzheimer’s disease that can cause this patient’s problem?”

Yes, there is a form of atypical Alzheimer's disease known as **logopenic variant primary progressive aphasia (lvPPA)**, which could cause this patient's problems. LvPPA is a clinical subtype of primary progressive aphasia, and it is often associated with Alzheimer's disease pathology. It is characterized by progressive language impairment, including difficulties with word retrieval, sentence repetition, and phonemic errors, while memory and other cognitive functions may remain relatively preserved initially.

The patient's difficulties in writing, reading, naming objects, dyslexia, and dysgraphia, along with the relatively preserved memory, judgment, and executive function, are consistent with lvPPA. The presence of right homonymous hemianopia may also suggest an atypical presentation of Alzheimer's disease or another underlying neurodegenerative process.

In this case, it would be essential to perform a comprehensive neurological workup, including neuropsychological testing and brain imaging (such as MRI or FDG-PET), to evaluate for potential Alzheimer's disease pathology and confirm the diagnosis of lvPPA. A consultation with a neurologist or speech and language therapist may also help guide the diagnostic process and management of the patient's language difficulties.

**Patient 6**

For the following medical history, we would like to ask you some questions at the end.

She was a 73 year old woman referred for work-up of decline in cognitive function over a 3 week period. She was admitted because of reduced general condition and mobility over a three week period with prominent memory impairment and repeatedly searching for things and no longer able to perform housework. She had reduced oral intake and with repeated crying episodes. Her husband recently passed away. There was no history of fever. Past medical history included hypertension, hyperlipidemia, chronic renal failure with creatinine around 220 umol/L, eGFR of 17 ml/min with proteinuria and history of osteoporotic vertebral collapse.

Physical examination was unremarkable. Urgent plain computed tomography of the brain was unremarkable, without any evidence of acute stroke. Vitamin B12, folate, thyroid function test and Venereal Disease Research Laboratory were unremarkable. Complete blood picture showed no evidence of leukocytosis. Urine culture was unremarkable. Blood biochemistries were unremarkable. Chest X-ray showed no evidence of pneumonia. Lumbar puncture was performed and no evidence of intracranial infection could be identified. During the hospital stay, she remained to have poor oral intake. Speech therapist noted dysphagia and Ryles’ tube was inserted for feeding.

Based on the above,

1. What is your primary diagnosis?
2. What are your differential diagnoses?
3. How would you work up for this patient?

GPT-4 reply:

1. Primary diagnosis:

Based on the provided history, the primary diagnosis for this 73-year-old woman is likely **major neurocognitive disorder** (also known as dementia). The patient's symptoms include a decline in cognitive function over a short period, memory impairment, reduced general condition, and mobility issues. These symptoms, along with the patient's age, are consistent with a neurocognitive disorder. Additionally, her recent bereavement (loss of her husband) could have exacerbated her cognitive decline.

2. Differential diagnoses:

Several conditions should be considered in the differential diagnosis, including:

- **Depression**: The patient's recent bereavement, crying episodes, and reduced oral intake could suggest depression, which can cause cognitive decline and mimic dementia.

- **Delirium**: The patient's acute onset of symptoms and fluctuating course may also indicate delirium, which can occur due to multiple medical conditions, including infections, metabolic disturbances, and medication side effects.

- **Nutritional deficiencies**: Although the tests for vitamin B12 and folate were unremarkable, other nutritional deficiencies could contribute to cognitive decline.

- **Normal pressure hydrocephalus**: This condition can cause gait disturbances, cognitive decline, and urinary incontinence. A brain CT scan was unremarkable, but further imaging (e.g., MRI) could provide more information.

3. Workup for this patient:

A comprehensive workup should aim to identify potential reversible causes of the patient's cognitive decline and any underlying conditions that may require treatment. Steps may include:

- **Neuropsychological testing**: A detailed cognitive assessment can help confirm the diagnosis of dementia and rule out other conditions like depression or delirium.

- **MRI of the brain**: This can provide additional information about the patient's brain structure and identify any abnormalities not detected on the CT scan.

- **Electroencephalogram (EEG)**: This may help identify any seizure activity or other abnormalities in brain function.

- **Blood tests**: Additional blood tests might be helpful to identify underlying metabolic disturbances, inflammation, or other potential causes of the patient's symptoms.

- **Medication review**: Reviewing the patient's current medications for potential side effects or interactions that may contribute to her cognitive decline.

- **Psychiatric evaluation**: A thorough evaluation of the patient's mental health can help determine if depression or other psychiatric conditions are contributing to her symptoms.

- **Social support evaluation**: Assessing the patient's social support network may help identify any additional resources or interventions that can improve her quality of life and cognitive functioning.

**Patient 7**

For the following medical history, we would like to ask you some questions at the end.

A 66 years old man has been followed up in memory clinic with amnesic mild cognitive impairment. He was initially referred because of one year history of impaired short term memory. Upon questioning, he was still able to recall the food that he ate in the last meal and able to recall the transport which he had taken to come here. He found it difficult to learn new things or concentrate on studying. The long term memory is preserved. His language was not impaired. He has preserved temporal and spatial orientation. He had no history of getting lost. However, he commented that his judgment has impaired. His basic activities of daily living were preserved. He could still handle money, handle his own banking, and operate on computer or mobile phone. His wife noted his temper was bad easily. He described his sleep as poor. He still enjoyed reading, swimming and had dim sum. His appetite was good. He has retired from property investment work. He received education up to master degree in business. His father suffered from dementia around his seventies. He was a non-smoker and non-drinker. He has no known drug allergy.

Physical examination was unremarkable. His blood pressure was 142/66 mmHg with body mass index of 38.4 kg/m2. Hong Kong version of the Montreal Cognitive Assessment was 22 out of 30. Magnetic resonance imaging was unremarkable. Single photon emission computed tomography showed no evidence of hypoperfusion in brain parenchyma. He described recently that he had more difficulty in concentration in the daytime and dozed off in the daytime.

Based on the above,

1. What is your primary diagnosis?
2. What are your differential diagnoses?
3. How would you work up for this patient?

GPT-4 reply:

1. Primary diagnosis:

Based on the provided information, the primary diagnosis for this 66-year-old man would be amnesic mild cognitive impairment (aMCI). He presents with a history of impaired short-term memory, difficulty in learning new things, and impaired judgment, but preserved long-term memory, language, and orientation. His basic activities of daily living are preserved, and he has a family history of dementia.

2. Differential diagnoses:

- Alzheimer's disease: Although the patient has aMCI, which may progress to Alzheimer's disease, currently he does not meet the criteria for Alzheimer's due to preserved daily functioning and absence of significant cognitive decline in other domains.

- Vascular cognitive impairment: This patient has hypertension and obesity, which are risk factors for vascular cognitive impairment. However, the absence of focal neurological signs and normal SPECT imaging make it less likely.

- Sleep apnea: The patient reports poor sleep and daytime sleepiness, which could be due to sleep apnea. This may contribute to cognitive impairment.

- Depression: Cognitive impairment can be a symptom of depression, and the patient reports irritability and poor sleep. However, the preserved interest in hobbies and good appetite make depression less likely in this case.

3. Workup for this patient:

- Comprehensive blood tests: Screen for metabolic and endocrine disorders, including complete blood count, electrolytes, liver and kidney function tests, thyroid function tests, and vitamin B12 and folate levels.

- Neuropsychological testing: Perform a detailed cognitive evaluation to assess the extent and severity of cognitive impairment and identify any other affected cognitive domains.

- Sleep study: Conduct a sleep study to evaluate for sleep apnea, which can contribute to cognitive impairment.

- Follow-up imaging: Repeat MRI or consider a PET scan to assess for amyloid or tau deposition in the brain, which may suggest the progression of aMCI to Alzheimer's disease.

- Monitor cognitive function over time: Regular follow-up visits to assess the progression of cognitive impairment and adjust the management plan accordingly.

**Patient 8**

For the following medical history, we would like to ask you some questions at the end.

A 64 years old man was referred to memory clinic for further work-up.

He was noted by his wife to have two years history of short term memory impairment, including repeated questioning, inability to locate his own objects, inability to recall recently occurred events & conversations, repeatedly washed dishes & windows, forgot to have meals and ate frequently. He was also unable to recall the names of relatives & name common objects. He had poor judgment and was unable to handle financial matters & even small sum of money. He showed impairment in activities of daily living with dressing apraxia, deterioration in skills of household works and inability to handle banking activities. He was apathetic and sleep excessively. He was suspicious of his maid stealing his things, repeated pacing at home and could get easily agitated. He received education up to secondary level three. He is a non-smoker and non-drinker. He previously worked as hotel manager and has retired. He had a past medical history of hyperlipidemia.

Physical examination including neurological examination was unremarkable. Mini-Mental State Examination Scores was 9 out of 30. Geriatric Depression Score was 1 out of 30. Vitamin B12, folate, thyroid function test and Venereal Disease Research Laboratory were negative. Computed tomography of the brain was unremarkable.

Based on the above,

1. What is your primary diagnosis?
2. What are your differential diagnoses?
3. How would you work up for this patient?

GPT-4 reply:

1. Primary Diagnosis:

Based on the patient's history, age, and clinical presentation, the primary diagnosis is likely Alzheimer's disease or another form of dementia. The patient exhibits short-term memory impairment, difficulty with activities of daily living, apraxia, and changes in personality and behavior, all of which are consistent with Alzheimer's disease.

2. Differential Diagnoses:

Some differential diagnoses to consider include:

- Vascular dementia: Cognitive decline due to cerebrovascular disease or multiple small infarcts in the brain.

- Lewy body dementia: Dementia with visual hallucinations, parkinsonian symptoms, and fluctuating cognition.

- Frontotemporal dementia: Dementia with changes in personality, behavior, and language dysfunction.

- Normal pressure hydrocephalus: Dementia with gait disturbance and urinary incontinence.

- Depression: Also known as pseudodementia, depression can cause cognitive symptoms that mimic dementia.

3. Workup for the Patient:

A thorough workup can help to confirm the primary diagnosis and rule out other potential causes. The workup may include:

- Comprehensive history and physical examination: A detailed history of the patient's symptoms, progression, and risk factors, as well as a thorough physical and neurological examination.

- Cognitive assessment: Formal neuropsychological testing to evaluate memory, language, attention, and other cognitive domains.

- Laboratory tests: Complete blood count, electrolytes, renal and liver function tests, and vitamin B12 and folate levels to rule out other potential causes of cognitive decline.

- Neuroimaging: Although the patient's CT scan was unremarkable, magnetic resonance imaging (MRI) may provide additional information about brain structure and help rule out other causes of cognitive decline, such as vascular dementia or brain tumors.

- Cerebrospinal fluid (CSF) analysis: Lumbar puncture for CSF analysis to assess for biomarkers of Alzheimer's disease, such as beta-amyloid and tau proteins.

- Genetic testing: If there is a strong family history of dementia, genetic testing for known dementia-related genes (e.g., APOE) may be considered.

Ultimately, the patient should be referred to a neurologist or geriatrician with expertise in cognitive disorders for further evaluation and management.

**Patient 9**

For the following medical history, we would like to ask you some questions at the end.

A 67 years old man was referred to memory clinic because of six months history of visual hallucination.

Regarding his vivid visual hallucination, he saw several persons were seen climbing outside the window of his flat, several strangers were yelling inside his house and persons inside a photo were talking to him. He showed fluctuation in his cognition and mental state. He sometimes slept for the whole day, sometimes very smart and awake. He was also found to have memory problems, including inability to recall the period of previous chemotherapy, misplacement of objects, repeated questioning and inability to recall previous conversation. He has been seen by his wife to have some abnormal behavior during sleep, namely throwing his arms and yelling around midnight after falling asleep in the middle of the night. Basic and instrumental activities of daily living remained independent. He has history of follicular lymphoma with complete remission, chronic hepatitis B on entecavir and psoriasis. He was a retired sales representative. He received education up to secondary level three. He was a non-smoker and non-drinker.

Physical examination was unremarkable. Parkinsonism was not noted. His Hong Kong Version of Montreal Cognitive Assessment was 16 out of 30. Computed tomography of the brain was unremarkable. Vitamin B12, folate, thyroid function test and Venereal Disease Research Laboratory were negative.

Based on the above,

1. What is your primary diagnosis?
2. What are your differential diagnoses?
3. How would you work up for this patient?

GPT-4 reply:

1. Primary diagnosis:

Based on the patient's age, history of visual hallucinations, fluctuations in cognition and mental state, memory problems, and abnormal behavior during sleep, the primary diagnosis is likely to be Lewy body dementia (LBD). LBD is a progressive neurodegenerative disease that affects cognition, behavior, and motor function.

2. Differential diagnoses:

- Alzheimer's disease: The patient's memory problems and cognitive decline may be suggestive of Alzheimer's disease. However, the presence of visual hallucinations and fluctuations in cognition are more characteristic of LBD.

- Parkinson's disease dementia (PDD): PDD shares some symptoms with LBD, such as cognitive decline and motor symptoms, but the patient does not exhibit any signs of parkinsonism.

- Delirium: The patient's fluctuating mental state may suggest delirium, but this is less likely given the chronic nature of his symptoms.

- Vascular dementia: This type of dementia can be caused by cerebrovascular disease, but the CT scan of the brain was unremarkable.

- Normal pressure hydrocephalus (NPH): NPH can present with cognitive and gait disturbances, but there is no mention of gait abnormalities or the classic triad of symptoms (cognitive decline, urinary incontinence, and gait disturbance) in the patient's history.

3. Workup for this patient:

- Detailed neuropsychological testing: To further assess the patient's cognitive function and identify specific areas of impairment.

- Magnetic resonance imaging (MRI): To evaluate for possible structural brain abnormalities, such as atrophy or white matter changes, that may not be apparent on CT.

- Electroencephalogram (EEG): To assess for any seizure activity or other abnormalities that may be contributing to the patient's symptoms.

- Polysomnography: To investigate the patient's sleep behavior and assess for any sleep disorders, such as REM sleep behavior disorder, which is often associated with LBD.

- DaTscan (Ioflupane I-123 injection): A dopamine transporter imaging agent that can help differentiate LBD from other types of dementia, as it can show reduced dopamine transporter uptake in the striatum in patients with LBD.

- Cerebrospinal fluid (CSF) analysis: To rule out other potential causes of dementia, such as infections or inflammation.

- Further blood work: To rule out any other metabolic or systemic causes of the patient's symptoms, such as liver or kidney dysfunction, electrolyte imbalances, or autoimmune diseases.

It is important to involve a multidisciplinary team, including neurologists, geriatricians, and neuropsychologists, in the evaluation and management of this patient.

**Patient 10**

For the following medical history, we would like to ask you some questions at the end.

An 84 years old woman was referred to memory clinic because of  two years history of short term memory impairment.

She has been observed to have two years history of short-term memory impairments, mainly with repeated questioning with marked temporal disorientation. She was noted have dysexecutive syndrome with a decrease ability to handle household activities, and impaired calculation. She was apathetic and had less motivation in going out for social interaction. She has a stereotyped behavior of buying a particular brand of bread (refusing to consume other brands of bread). The buying behavior was so abnormal that that brand of bread was sold out. She had no problem in recalling shopping list. She was illiterate and was a non-smoker and non-drinker. She had a history of hypertension, bilateral retinal detachment, cataract, and convergent squint.

Physical examination was unremarkable. Her Mini-Mental State Examination Score was 11 out of 30. Computed tomography of the brain was described as normal. Vitamin B12, folate, thyroid function test and Venereal Disease Research Laboratory were negative.

Based on the above,

1. What is your primary diagnosis?
2. What are your differential diagnoses?
3. How would you work up for this patient?

GPT-4 reply:

1. Primary Diagnosis:

Based on the patient's age, history, and presentation, the primary diagnosis would be Alzheimer's disease or another form of dementia. The patient's short-term memory impairment, dysexecutive syndrome, apathy, decreased social interaction, and stereotyped behavior are all classic symptoms of Alzheimer's disease or dementia.

2. Differential Diagnoses:

- Vascular dementia: This could be a possibility given the patient's history of hypertension and retinal detachment. Vascular dementia occurs due to reduced blood flow to the brain, leading to cognitive impairments.

- Frontotemporal dementia: This condition can cause similar symptoms as Alzheimer's disease but usually begins at a younger age and progresses more rapidly.

- Normal pressure hydrocephalus: This condition is characterized by an accumulation of cerebrospinal fluid in the brain's ventricles, causing cognitive and motor impairments.

- Mild cognitive impairment: This is a less severe form of cognitive decline that does not meet the criteria for dementia.

3. Workup for this patient:

- Neuropsychological testing: Assess the patient's cognitive function in more detail, including memory, language, executive function, and visuospatial skills.

- Blood tests: Recheck Vitamin B12, folate, and thyroid function tests to rule out any reversible causes of cognitive decline.

- Brain imaging: Magnetic resonance imaging (MRI) or positron emission tomography (PET) scan can be helpful in evaluating structural and functional changes in the brain, as well as to rule out other possible causes of cognitive decline.

- Cerebrospinal fluid (CSF) analysis: Lumbar puncture can be performed to analyze CSF for biomarkers related to Alzheimer's disease or other neurodegenerative disorders.

- Consultation with a neurologist, geriatrician, or psychiatrist: A specialist may provide further insights and recommendations for diagnosis and management.

It is essential to involve the patient's family in the diagnostic process and discuss any findings and recommendations with them. Early diagnosis and intervention can help manage the symptoms and potentially slow the progression of the disease.
