# Supplementary material for: Limitations of GPT‐4 in analyzing real‐life medical notes related to cognitive impairment
Source: Psychogeriatrics. 2023 Jun 27;23(5):885–7. doi: 10.1111/psyg.13002 (PMC11577998; doi:10.1111/psyg.13002)
Supplement: Supplementary file 2 — Data S2. The neuroimaging findings supporting the diagnoses of our cases series. [file PSYG-23-885-s001.docx]

**Supplementary file 2**

The neuroimaging findings supporting the diagnoses of our cases series

**Patient 2 (corticobasal syndrome)**


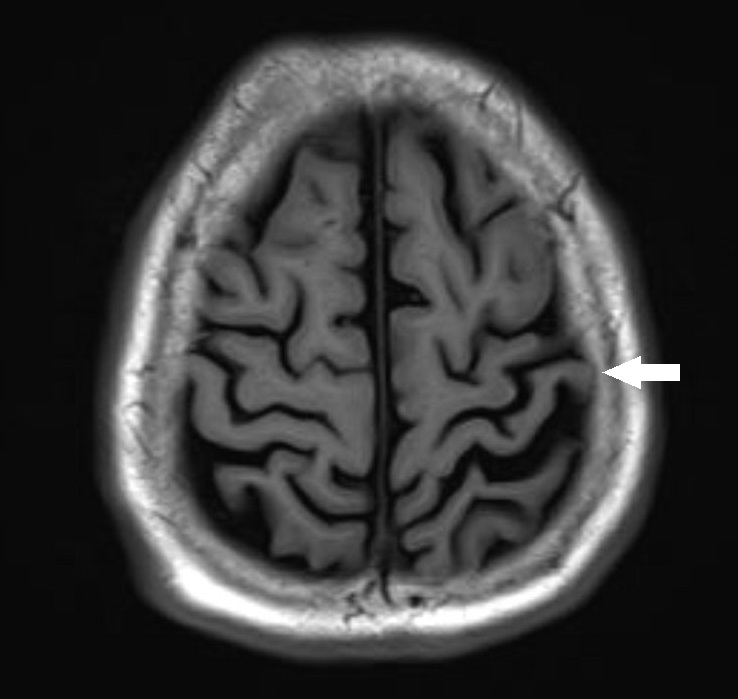


MRI brain T1 sequence showing the left perirolandic atrophy (white arrow)^1^

**Patient 3 (semantic dementia)**

**
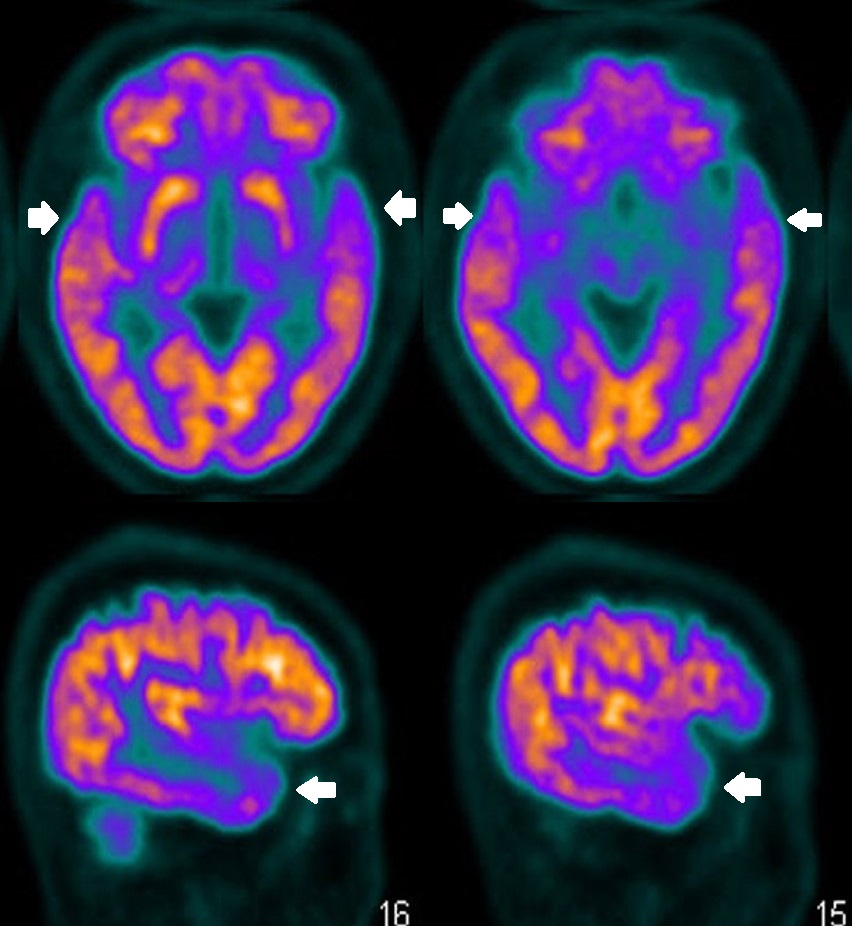
**

^18^FDG-PET showing the bilateral anterior temporal lobes hypometabolism (white arrows)^2^

**Patient 4 (corticobasal syndrome)**


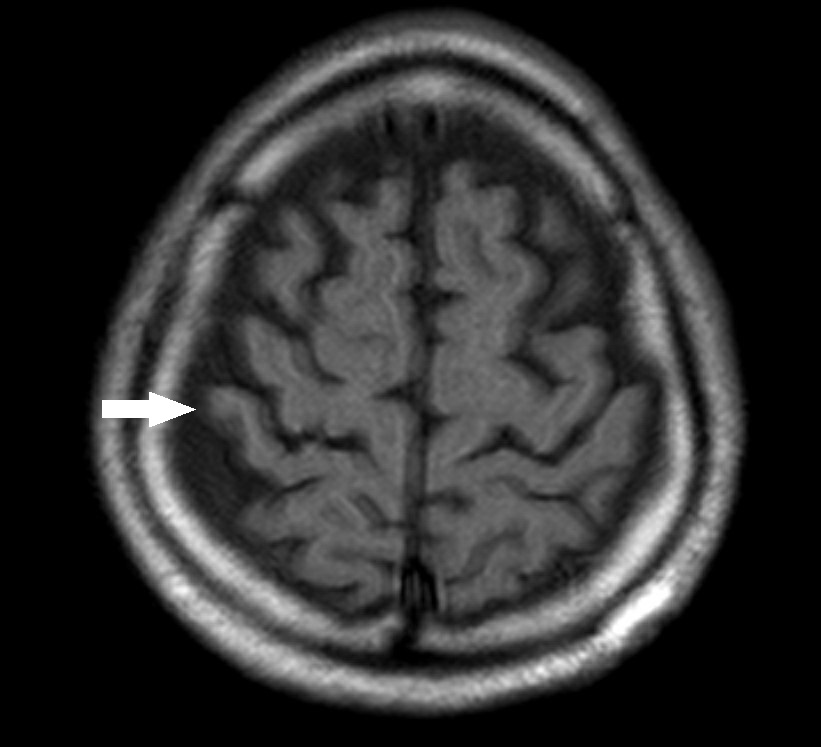


MRI brain T1 sequence showing the right perirolandic atrophy (white arrow)^1^

**Patient 6 (Creutzfeldt Jakob disease)**


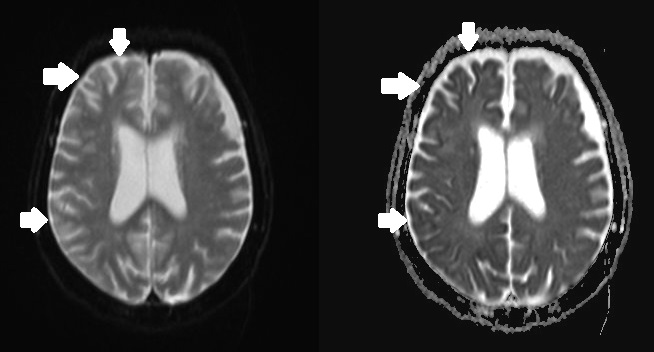


Diffusion weighted imaging MRI on the left side showing the diffuse cortical ribboning (i.e. hyperintensities) (white arrows) with corresponding suppression on apparent diffusion coefficient mapping on the right side (white arrows) reflecting the restricted water diffusivity.^4^

**Patient 9 with dementia with Lewy Bodies**


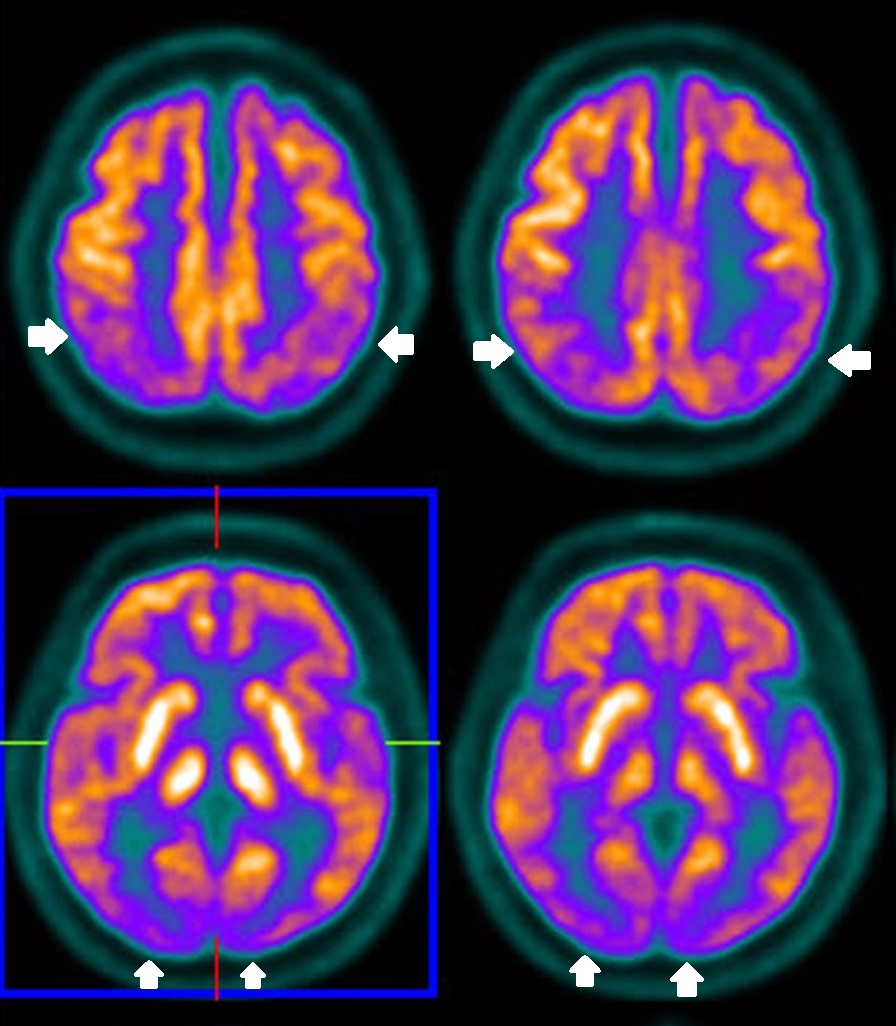


^18^FDG-PET showing the bilateral parieto-occipital lobes hypometabolism (white arrows).^4^

**Patient 10 – behavioral variant of frontotemporal dementia**


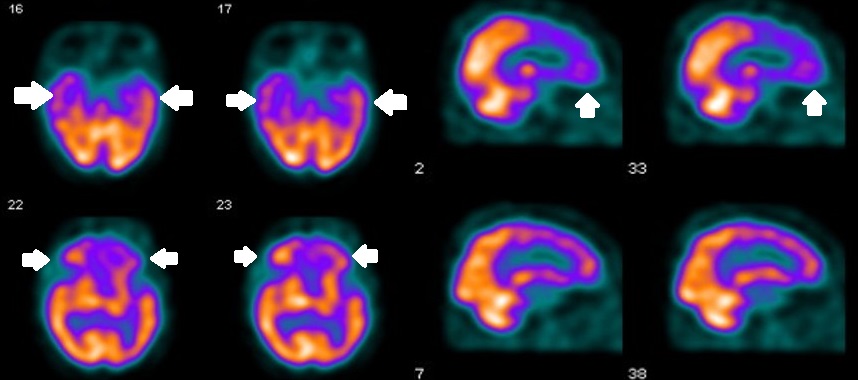


Single photon emission computed tomography of the brain showing the bilateral frontotemporal hypoperfusion (white arrows).^3^

**References:**

1. Constantinides VC, Paraskevas GP, Paraskevas PG, Stefanis L, Kapaki E. Corticobasal degeneration and corticobasal syndrome: A review. *Clin Park Relat Disord* 2019;**1**:66-71.

2. Finger EC. Frontotemporal Dementias. *Continuum (Minneap Minn)* 2016;**22**:464-89.

3. Hermann P, Appleby B, Brandel JP, Caughey B, Collins S, Geschwind MD, et al. Biomarkers and diagnostic guidelines for sporadic Creutzfeldt-Jakob disease. *Lancet Neurol* 2021;**20**:235-46.

4. Walker Z, Possin KL, Boeve BF, Aarsland D. Lewy body dementias. *Lancet* 2015;**24**:1683-97.
